# Genetic Determinants and Prediction of Antibiotic Resistance Phenotypes in *Helicobacter pylori*

**DOI:** 10.3390/jcm8010053

**Published:** 2019-01-07

**Authors:** Francis N. Lauener, Frank Imkamp, Philippe Lehours, Alice Buissonnière, Lucie Benejat, Reinhard Zbinden, Peter M. Keller, Karoline Wagner

**Affiliations:** 1 Institute of Medical Microbiology, University of Zurich, 8006 Zurich, Switzerland; francisnicolai.lauener@uzh.ch (F.N.L.); imkamp@imm.uzh.ch (F.I.); rzbinden@imm.uzh.ch (R.Z.); pkeller@imm.uzh.ch (P.M.K.); 2 INSERM UMR1053, Bordeaux Research in Translational Oncology, BaRITOn, Université de Bordeaux, 33076 Bordeaux, France; philippe.lehours@u-bordeaux.fr (P.L.); alice.buissonniere@chu-bordeaux.fr (A.B.); lucie.bruhl@chu-bordeaux.fr (L.B.); 3 French National Reference Centre for Campylobacter and Helicobacter, Bordeaux Hospital, 33076 Bordeaux, France; 4 Institute for Infectious Diseases, University of Bern, 3001 Bern, Switzerland

**Keywords:** *Helicobacter pylori*, whole genome sequencing, antibiotic resistance prediction, phenotypic drug susceptibility testing, laboratory automation

## Abstract

*Helicobacter pylori* is a major human pathogen. Diagnosis of *H. pylori* infection and determination of its antibiotic susceptibility still mainly rely on culture and phenotypic drug susceptibility testing (DST) that is time-consuming and laborious. Whole genome sequencing (WGS) has recently emerged in medical microbiology as a diagnostic tool for reliable drug resistance prediction in bacterial pathogens. The aim of this study was to compare phenotypic DST results with the predictions based on the presence of genetic determinants identified in the *H. pylori* genome using WGS. Phenotypic resistance to clarithromycin, metronidazole, tetracycline, levofloxacin, and rifampicin was determined in 140 clinical *H. pylori* isolates by E-Test^®^, and the occurrence of certain single nucleotide polymorphisms (SNPs) in target genes was determined by WGS. Overall, there was a high congruence of >99% between phenotypic DST results for clarithromycin, levofloxacin, and rifampicin and SNPs identified in the 23S rRNA, *gyrA*, and *rpoB* gene. However, it was not possible to infer a resistance phenotype for metronidazole based on the occurrence of distinct SNPs in *frxA* and *rdxA*. All 140 *H. pylori* isolates analysed in this study were susceptible to tetracycline, which was in accordance with the absence of double or triple nucleotide substitutions in the 16S rRNA gene.

## 1. Introduction

*Helicobacter pylori* is a gram-negative bacterium that infects about half of the world’s human population. Unless treated, colonization in the human gastrointestinal tract persists lifelong. *H. pylori* infection represents a key factor in the aetiology of various gastroduodenal diseases, including chronic active gastritis, peptic or duodenal ulcers, gastric adenocarcinoma, and mucosa associated tissue lymphoma [1,2,3]. The current first-choice empiric regimen for *H. pylori* eradication in countries with clarithromycin resistance <15% consists of a clarithromycin-based triple therapy with a proton pump inhibitor (PPI) in combination with metronidazole or amoxicillin [4,5]. However, in the last years, the effectiveness of this empiric first-line therapy has been steadily reduced due to increasing clarithromycin and/or metronidazole resistance in *H. pylori* [6,7,8]. Alternatively, non-bismuth or bismuth containing quadruple therapy combined with tetracycline, levofloxacin, or rifabutin-based antibiotic regimens can be administered to patients [5,9]. However, the increase in quinolone resistant *H. pylori* strains [10,11], and the emergence of quadruple-resistant *H*. *pylori* clinical isolates in Western Europe [8,12] emphasize the need for more rapid and cost effective molecular methods that enable reliable prediction of antibiotic resistance phenotypes prior to the administration of antimicrobial therapy.

Diagnosis of *H. pylori* infection and determination of the pathogen’s antibiotic susceptibility mainly relies on bacterial culture and phenotypic drug susceptibility testing (DST), typically delivering results within two weeks. However, visual inspection of minimum inhibitory concentrations (MICs) on the E-Test^®^ strips leaves great scope for personal interpretation and potentially leads to high inter-observer variability due to the fastidious growth of *H. pylori* [13].

Recently, whole-genome sequencing (WGS) from cultured bacterial isolates has emerged as an important tool for surveillance and antibiotic resistance control. This primarily owes to improvements in sequencing technologies, affordable instrument pricing, user friendly and simple workflows requiring little hands on time, availability of standardized protocols and reagents, and reasonable per sample costs (150 EUR per 5 MB genome in the case of the Illumina MiSeq), and are hence an attractive choice as a diagnostic tool to detect drug resistance in diagnostic microbiology laboratories. Within a clinically relevant timeframe (24 to 48 h), WGS provides a comprehensive view of the genotype of a bacterial isolate [14,15]. In principle, the genome sequence contains all the information required to determine the antibiotic resistance phenotype. The biggest challenge, however, lies in the identification of high-confidence single nucleotide polymorphisms (SNPs) associated with resistance against a certain drug. Genotype-based prediction of phenotypic resistance is less complex for *H. pylori* than for other bacteria as antibiotic resistance is primarily based on point mutations in the genome and resistance determinants in *H. pylori* seem not to be encoded on plasmids, transposons, or integrons [16].

In detail, clarithromycin resistance in *H. pylori* has been associated with point mutations in domain V of the 23S rRNA gene, namely at the nucleotide position, A2146 and A2147 (positions according to *H. pylori* reference strain 26695; correspond to nucleotides A2058 and A2059 in *Escherichia coli*) [17]. Some studies have reported point mutations outside these positions, but their association with clarithromycin resistance is still a matter of debate [18,19,20]. Amino acid (aa) exchanges in the quinolone resistance-determining region (QRDR) of the *H. pylori *gyrA** gene at codons 87 and 91 (positions according to *H. pylori* reference strain 26695; corresponds to codons 83 and 87 in *Escherichia coli*) alone or in combination with mutations in *gyrB* have been reported to lead to resistance to fluoroquinolones [10,11,21]. Rifampicin resistance in *H. pylori* has been associated with aa exchanges in the rifampicin resistance-determining region (RRDR) of *rpoB*, mainly at codons 525 to 545, 547, and 586 (positions according to *H. pylori* reference strain 26695; corresponds to codons 512 to 573 in *Escherichia coli*) [22]. The mechanism of resistance to metronidazole is less clear. Frameshift mutations and truncations in genes that encode electron transfer proteins, such as the nicotinamide adenine dinucleotide phosphate hydrogen (NADPH)-flavin nitroreductase (*frxA*) and oxygen-insensitive NADPH nitroreductase (*rdxA*), have been implicated in elevated MIC values to metronidazole [23,24]. Thus far, it is unclear, whether elevated metronidazole MIC values lead to therapeutic failure [25]. For tetracycline, one resistance mechanism appears to be single, double, or triple base pair substitutions in the primary binding site of tetracycline (at nucleotide positions 926 to 928) in the 16S rRNA gene [26].

Here, we report on the first sequencing study that systematically applied WGS to *H. pylori* clinical isolates to detect specific point mutations in the 23S rRNA, *gyrA*, *rpoB*, *frxA*, *rdxA*, and 16S rRNA genes. We aimed at correlating the occurrence of SNPs in these target genes to phenotypic DST results for each drug investigated, thereby allowing the calculation of predictive sensitivities and specificities of each set of SNPs.

## 2. Materials and Methods

### 2.1. Clinical H. pylori Isolates and H. pylori Culture

This study was conducted using a set of 140 *H. pylori* strains from the bacterial strain collection of the Institute of Medical Microbiology (IMM), University of Zurich. The *H. pylori* strains were isolated between 2013 and 2017 from gastric biopsy specimens that were sent to the IMM for culture-based phenotypic DST. For this study, *H. pylori* strains were intentionally selected based on their antimicrobial resistance phenotype and are therefore not representative of *H. pylori* primary antibiotic resistance epidemiology in Switzerland. After thawing, the strains were incubated on in-house produced *Brucella* agar plates (with 5% horse blood) for 3 days at 37 °C under microaerobic conditions (90% N_2_, 5% CO_2_, 5% O_2_) using a gas generator (CampyGen, Thermo Scientific, Waltham, MA, USA). After 3 days, *H. pylori* isolates were subcultured on *Brucella* agar plates to obtain sufficient biomass for the E-Test^®^ (bioMérieux, Marcy l’Etoile, France) and to perform DNA extraction for WGS.

### 2.2. Phenotypic DST by E-Test^®^

**Figure 1:**
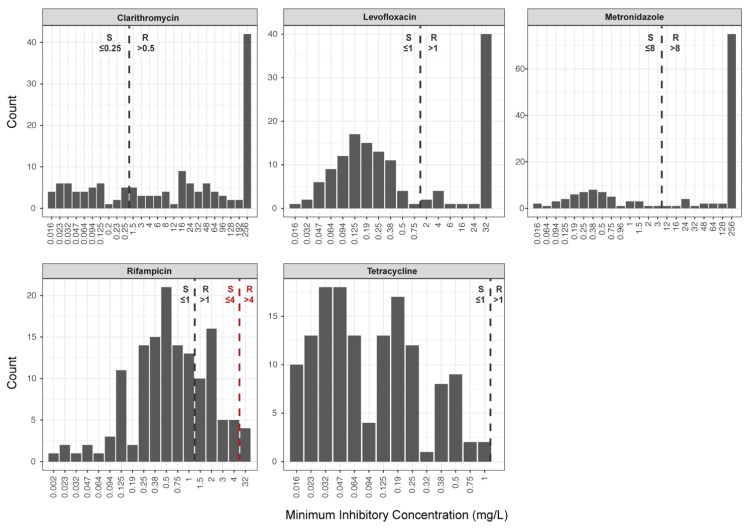
Antimicrobial drug susceptibility determined by E-Test^®^ in 140 clinical *H. pylori* isolates selected from the bacterial strain collection of the Institute of Medical Microbiology, University of Zurich, Switzerland. Clinical breakpoints issued by EUCAST [28] are displayed for all five drugs (black dashed line). For rifampicin, the clinical breakpoint of 4 mg/L [22,29] is displayed as a red dashed line; R denotes resistant and S denotes susceptible.

*H. pylori* cultures were adjusted to a McFarland standard of 3 [27]. Phenotypic DST was performed on Mueller Hinton agar plates containing 5% horse blood (bioMérieux), using the following E-Tests^®^ (bioMérieux): Clarithromycin (0.016–256 mg/L), metronidazole (0.016–256 mg/L), levofloxacin (0.016–32 mg/L), rifampicin (0.016–32 mg/L), and tetracycline (0.016–32 mg/L). Agar plates were incubated under microaerobic conditions at 37 °C for 3 days. Subsequently, MICs were determined using a light microscope (Leica M80, Leica Microsystems, Heerbrugg, Switzerland). Susceptibility interpretation was performed according to the European Committee on Antimicrobial Susceptibility Testing (EUCAST) [28], except for rifampicin, for which the susceptibility interpretation (MIC: S ≤ 4; R > 4) was done according to Hays et al. [22] and the Comité de l’Antibiogramme de la Société Française de Microbiologie (CASFM)/EUCAST recommendations for *H. pylori* [29] (Figure 1).

### 2.3. DNA Extraction, Library Preparation, and WGS of H. pylori Strains

DNA extraction from *H. pylori* cultures was performed with the DNeasy^®^ UltraClean^®^ Microbial kit (Qiagen, Hilden, Germany), following the producers’ recommendations. Library preparation was done using the Qiagen^®^ QIAseq FX DNA kit (Qiagen, Hilden, Germany), according to the producers’ recommendations. Sequencing library quality and size distribution were analysed on a fragment analyser automated CE system (Advanced Analytical Technologies Inc., Heidelberg, Germany), according to the manufacturers’ instructions using the fragment analyser 474 HS next generation sequencing (NGS) kit. Sequencing libraries were pooled in equimolar concentrations and paired-end sequenced (2 × 150 bp) on an Illumina MiSeq platform (Illumina^®^, San Diego, CA, USA).

### 2.4. Bioinformatic Analysis

Raw sequencing reads (fastq) were filtered and trimmed using the FASTQ trimmer tool of the FASTX-Toolkit (Hannon Lab, Cold Spring Harbour Laboratories, Cold Spring Harbor, NY, USA) applying a threshold PHRED score of 25. To identify SNPs in genes conferring resistance, fastq files were analysed using the ARIBA pipeline [30], querying a custom-made database of gene sequences derived from the *H. pylori* reference strain 26695 (NCBI reference sequence: NC_000915.1). For the evaluation of metronidazole resistance, all SNPs in *rdxA*, *frxA*, *mdaB*, *omp11*, and *rpsU* that occurred more frequently in metronidazole resistant than susceptible *H. pylori* isolates were assessed. 

In order to identify probable plasmid-encoded resistance genes, trimmed and filtered FASTQ sequencing files were analysed using the plasmidSPAdes pipeline (Version 3.10.1; [31]). Obtained contigs were used to perform a blastn search querying the NCBI Nucleotide database.

### 2.5. Discrepancy Analysis

*H. pylori* isolates showing discrepant phenotypic DST and WGS results were sent to the French National Reference Centre for *Campylobacter* and *Helicobacter* (Bordeaux, France) (www.cnrch.fr) for repetition of phenotypic DST. Briefly, *H. pylori* isolates were adjusted to a McFarland of 3 and grown on in-house produced Mueller Hinton agar plates (with 10% sheep blood and globular extract) for 48 h at 35 °C in a microaerobic atmosphere (5% O_2_, 10% CO_2_, and 85% N_2_) in a special workstation (Baker, Ruskin, Concept, Bridget, UK). After 48 h, MICs were determined by E-Test^®^ (bioMérieux), and antibiotic susceptibilities were assessed based on the CASFM/EUCAST recommendations for *H. pylori* [29].

In addition, all discrepant *H. pylori* strains were grown on in-house produced *Brucella* agar plates (with 5% horse blood), and DNA extraction for WGS was repeated at the Institute of Medical Microbiology, Zurich, Switzerland.

### 2.6. Statistics and Data Analysis

We used the 2 × 2 contingency table to calculate the agreement between WGS and phenotypic DST results [32,33]. In this context, specificity describes the likelihood that SNPs identified in a target gene lead to phenotypic drug resistance, while sensitivity describes the proportion of resistant cases that are identified through that set of SNPs. A major error occurred if a *H. pylori* strain that was identified as susceptible by phenotypic DST was determined resistant by WGS. In this case, an alternative antimicrobial regimen would have been prescribed to the patient, though first line treatment could be administered. A very major error occurred if an *H. pylori* strain that was identified as resistant by phenotypic DST was determined susceptible by WGS. In this case, the organism would be falsely assessed as susceptible, resulting in the administration of an ineffective first-line antibiotic therapy. A chi-square test (*X^2^*) of independence was performed to examine the relationship between the occurrence of certain SNPs and the antibiotic resistance phenotype. All statistics and data visualization was done in R [34].

### 2.7. Declarations

#### 2.7.1. Ethics Approval and Consent to Participate

The study was conducted according to good laboratory practice and in accordance with the Declaration of Helsinki and national and institutional standards. The Swiss act on medical research involving human subjects does not apply to this study as solely bacterial strains and no human health-related data were used in this study. 

#### 2.7.2. Consent for Publication

No consent for publication is required. The Swiss act on medical research involving human subjects do not apply to this study as solely bacterial strains and no human health-related data were used.

## 3. Results

### 3.1. Occurrence of Plasmids Carrying Resistance Genes in H. pylori

In the 140 analysed *H. pylori* genomes, we did not detect a single plasmid encoding a resistance gene. This points towards the primary importance of SNPs in target genes in mediating phenotypic drug resistance in *H. pylori* and facilitates the prediction of resistance phenotypes.

### 3.2. Coherence in Prediction of Clarithromycin Resistance

Forty-three *H. pylori* strains showed a clarithromycin susceptible phenotype and, accordingly, displayed no mutations in domain V of the 23S rRNA gene at nucleotide position A2146 or A2147. Two of the 43 susceptible *H. pylori* isolates (with MICs of 0.016 and 0.023 mg/L) carried a G2224A mutation, which does not cause macrolide resistance [35]. 

One isolate carried a T2182C mutation and exhibited a low-level clarithromycin resistance with an MIC of 1.5 mg/L. As the relevance of the T2182C mutation in clarithromycin resistance is questionable [18], this strain was sent for retesting to the *H. pylori* reference centre in Bordeaux, where an MIC of 0.5 mg/L was determined (Table S1). Thus, initially, phenotypic DST falsely identified this *H. pylori* strain as clarithromycin resistant, representing a major error.

Two *H. pylori* strains initially showed an MIC of 0.19 and 0.5, but carried an A2146G and an A2147G mutation, respectively. Repetition of the E-Test^®^ eventually revealed a mixed population of clarithromycin susceptible and resistant *H. pylori* strains (0.19 and 6; 0.5 and >256). These two samples were scored as clarithromycin resistant *H. pylori* strains for further statistical analysis (Table 1 and Table 2).

Thus, in total, 96 *H. pylori* isolates were determined as clarithromycin resistant by phenotypic DST, and 28 A2146G (29%), 67 A2147G (70%), and one A2146C (1%) mutation were found (Table 1 and Table 2). Moreover, different point mutations resulted in different levels of clarithromycin resistance. While A2147G isolates showed MICs of 1.5 to >256, strains harbouring either the A2146G or the A2142C point mutation always displayed high clarithromycin resistance levels (MIC of 8 to ≥256 mg/L) (Table 1, Figure S1 Panel A).

Overall, the congruence between the phenotypic DST and WGS results was 99%.

Specificity, i.e., the likelihood that the presence of a mutation at nucleotide position A2146 and A2147 in the 23S rRNA gene resulted in a clarithromycin resistant phenotype, was 100%. Sensitivity, which describes the proportion of resistant isolates that were identified through the presence of a mutation at nucleotide positions, A2146 and A2147, was also 100%. Thus, the presence of point mutations, A2146C, A2146G, and A2147G, in the 23S rRNA gene of *H. pylori* could be significantly related to phenotypic clarithromycin resistance (*X*^2^ [1, *N* = 140] = 135.4, *p* < 0.001).

### 3.3. Coherence in Prediction of Metronidazole Resistance

Frameshift mutations and truncations in the genes, *rdxA* or *frxA*, are generally supposed to lead to metronidazole resistance in *H. pylori* [24]. Some frameshift mutations (occurring at codon positions, 105, 149, or 192, in *frxA* and 18, 38, and 112 in *rdxA*) were only detected in metronidazole resistant *H. pylori* isolates (Tables S2 and S3). Other frameshift mutations (occurring at codon positions 18 in *frxA* and 62, 96, and 162 in *rdxA*) occurred in both metronidazole susceptible and resistant *H. pylori* strains (Tables S2 and S3). SNPs resulting in the aa exchanges, A67V, A68E, K64N, P106S, and R16C, merely occurred in the *rdxA* gene of metronidazole resistant *H. pylori* strains (Table S4). All frameshift mutations and aa exchanges detected in *rdxA* and *frxA* of the *H. pylori* isolates and their corresponding metronidazole MIC are displayed in Tables S5 and S6. Interestingly, we did not detect any published SNPs associated with phenotypic metronidazole resistance within *mdaB*, *omp11*, or *rpsU* that were more prevalent in metronidazole resistant compared to susceptible *H. pylori* isolates.

### 3.4. Coherence in Prediction of Levofloxacin Resistance

In 90 of the 91 levofloxacin susceptible *H. pylori* isolates, the *gyrA* QRDR carried no SNP at codon 87 or 91. One *H. pylori* strain harboured an N87T aa exchange that has not been associated with levofloxacin resistance [11]. In 46 fluoroquinolone resistant *H. pylori* strains, WGS identified SNPs at codon 87 (*N* = 25; 54%), codon 91 (*N* = 19, 41%), and at codon 87 and 91 (*N* = 2; 5%) (Table 3 and Table 4). Single aa exchanges at codon 87 or 91 led to a relatively broad range of levofloxacin MICs (4 to 32 mg/L). High-level resistance (i.e., MIC > 32 mg/L) was exclusively found in the presence of aa exchanges at codons 87 and 91 (Table 3).

Three strains were phenotypically resistant to levofloxacin with MICs of 2 and 4 mg/L, respectively, but showed no SNPs in the *gyrA* QRDR (Figure S1, Panel B). Unfortunately, two of the three *H. pylori* strains showing discrepant results could not be regrown for discrepancy analysis. Thus, we were not able to verify the correctness of phenotypic DST or WGS and decided to subsequently exclude these two *H. pylori* strains from the study. The third *H. pylori* strain could be regrown and was sent for retesting to the *H. pylori* reference centre in Bordeaux. There, it showed an MIC of 1.5 mg/L (Table S1). The *gyrB* gene of this discrepant *H. pylori* isolate was analysed as well and showed multiple unpublished SNPs (compared to the *gyrB* sequence of the *H. pylori* reference strain 26695; Table S7).

The congruence between the phenotypic DST and WGS results was 99%. Specificity, i.e., the likelihood that aa exchanges in the QRDR of the *gyrA* gene at codon 87 and 91 confer levofloxacin resistance, was 100%. Sensitivity, i.e., the proportion of resistant cases that were identified through the presence of specific aa exchanges at codon 87 and 91 in the *gyrA* gene, was 98%. Overall, one major error occurred: One *H. pylori* isolate tested levofloxacin resistant by phenotypic DST was categorized as susceptible by WGS. Nevertheless, specific aa exchanges at codon 87 (N87I, N87K and N87Y) and 91 (D91N and D91Y) of the *gyrA* gene in *H. pylori* can be used to predict phenotypic levofloxacin resistance (*X*^2^ [1, *N* = 138] = 136.0, *p* < 0.001).

### 3.5. Coherence in Prediction of Rifampicin Resistance

All 136 rifampicin susceptible *H. pylori* isolates (MIC ≤ 4 mg/L) did not show an aa exchange in the RRDR of the *rpoB* gene. The four phenotypic resistant *H. pylori* isolates with MICs >32 mg/L had either an H540N (*N* = 2) or an L525P (*N* = 2) aa exchange (Table 5 and Table 6). Overall, an agreement of 100% was found between the WGS and phenotypic rifampicin susceptibility results.

### 3.6. Coherence in Prediction of Tetracycline Resistance

All 140 *H. pylori* strains included in this study were tetracycline susceptible in phenotypic DST. In two isolates with MICs of 0.25 and 0.5 mg/L, an A926T mutation was detected in the 16S rRNA gene. One *H. pylori* isolate showed an A926G mutation with an MIC of 0.032 mg/L, and one carried an A928C mutation with an MIC of 0.125 mg/L (Table 7). It has been previously reported that an A926G or an A928C nucleotide exchange was associated with a broad range of MICs, ranging from susceptible (0.75 mg/L) to low level resistance (4 mg/L), whereas double mutants displayed MICs of >4 mg/L [36,37].

## 4. Discussion

In the present study, we have applied massive parallel Illumina-based WGS on clinical *H. pylori* isolates to define the relationship between the occurrence of SNPs in selected target genes and phenotypic antimicrobial susceptibility. We could show a clear correlation between the occurrence of point mutations in the 23S rRNA, *gyrA*, and *rpoB* genes of *H. pylori* and macrolide, fluoroquinolone, and rifamycin resistance, respectively. To our knowledge, this is the first study showing that genetic determinants of antimicrobial resistance identified by WGS can be used for the prediction of drug resistance phenotypes in *H. pylori*. In contrast, there was no clear association between identified SNPs in *frxA* and *rdxA* and phenotypic metronidazole resistance.

During this study, we realized that assessment of antimicrobial susceptibility in *H. pylori* by culture based phenotypic DST can be challenging due to the pathogen’s fastidious growth requirements. Moreover, it was not always possible to determine an explicit MIC of a drug by E-Test^®^ because of the small and transparent colonies that *H. pylori* forms. Variations in the redox potential of the test medium caused a broad range of MIC distributions, especially for metronidazole, when performing the E-Test^®^, thereby making standardized and reproducible interpretation difficult. Phenotypic DST may also be challenged when a mixed population of resistant and susceptible *H. pylori* strains is present in the same patient [38]. In two samples included in this study, phenotypic DST initially did not detect the resistant sub-population. This is problematic, as antimicrobial therapy is based on in vitro phenotypic susceptibly information. In contrast, the presence of susceptible and resistant *H. pylori* subpopulations can be predicted from the WGS sequencing data through the occurrence of hetero-resistance at specific nucleotide positions [38].

Molecular based methods, like polymerase chain reaction (PCR) and line probe assays, that enable specific detection of point mutations in the 23S rRNA (nucleotide positions, A2146 and A2147) or *gyrA* gene (codons 87 and 91) can be directly applied on clinical specimens [39,40]. Our data indicate that molecular assays targeting these point mutations would be sufficient to monitor clarithromycin and levofloxacin resistance in *H. pylori*. However, one advantage of WGS is that it delivers a more complete picture of resistance determinants present in a clinical isolate than targeted molecular approaches that can only examine a limited number of nucleotide positions. Thus, false negative results may occur, when new polymorphisms, potentially conferring drug resistance, arise that are not covered by the assay. In contrast, the relevance of these polymorphisms can easily be assessed by retrospective analysis of WGS data, whereas targeted molecular assays would need to be redesigned and samples retested.

In this study, we found clarithromycin resistance in *H. pylori* to be highly correlated with the presence of mutations A2146C, A2146G or A2147G in the domain V of the 23S rRNA gene. Recent studies reported additional point mutations outside the domain V and an active drug efflux mechanism to be involved in clarithromycin resistance [35,41]. Also, mutations in other target genes, like *rpl22* (encodes a ribosomal protein that interacts with the 23S rRNA domains) and *infB* (encodes translation initiation factor, IF-2), were identified to induce low level clarithromycin resistance (MICs of 0.5 to 4 mg/L). Interestingly, these mutations led to a high level of clarithromycin resistance (MICs >256 mg/L) in combination with A2146 and A2147 mutations [42]. In a recent study, it was reported that mutations in multidrug efflux transporter genes may be involved in clarithromycin resistance [43]. In this study, however, we found that the occurrence of point mutations at nucleotide positions, A2146 and A2147, were sufficient for the reliable prediction of phenotypic clarithromycin resistance in *H. pylori*.

For metronidazole, there was no clear correlation between the isolates’ observed phenotype and their genotype. Firstly, frameshift mutations in *rdxA* and *frxA* that were reported to confer metronidazole resistance were also found in *H. pylori* strains assessed as metronidazole susceptible by phenotypic DST. Secondly, in contrast to recent reports suggesting that metronidazole resistance requires mutations in both genes, *frxA* and *rdxA*, one isolate was found to be resistant (MIC of 26 mg/L) though carrying only a mutation in *frxA* [44,45]. Some *rdxA* and *frxA* mutations were identified in metronidazole resistant *H. pylori* isolates; however, several metronidazole resistant strains harboured no mutations at all in *rdxA* and/or *frxA.* Recently, it has been reported that additional genes, such as *mdaB, omp11*, and *rpsU*, may be involved in metronidazole resistance in *H. pylori* [46,47]. However, consensual data are still lacking and a relationship between SNPs detected in one of these five genes (*rdxA*, *frxA*, *mdaB*, *omp11*, and *rpsU*) and phenotypic metronidazole resistance was not found in the *H. pylori* isolates investigated in this study. In sum, it remains difficult to predict metronidazole susceptibility based merely on genotypic data, as the correlation between detected SNPs and the phenotype to date is very poor.

On the basis of the presented data, we therefore suggest to reconsider the usefulness of phenotypic metronidazole susceptibility testing in *H. pylori* as it is stated in the CASFM/EUCAST recommendations for *H. pylori* [29]. Moreover, in vitro and in vivo resistance data are not congruent [48] as *H. pylori* strains tested resistant in vitro can still be eradicated with a combination therapy that contains an increased dosage of metronidazole [25].

Resistance to fluoroquinolones could be attributed to mutations in the QRDR of *gyrA* at codons 87 and 91 in 98% of the fluoroquinolone resistant *H. pylori* strains. One levofloxacin resistant *H. pylori* strain with an MIC of 1.5 mg/L showed no codon exchange in the QRDR of *gyrA*. The relevance of mutations in other target genes, like *gyrB*, in quinolone resistant *H. pylori* isolates without *gyrA* mutations is not consensual [49,50]. The aforementioned isolate harboured various SNPs in *gyrB*; however, none matched any mutations recently reported to be associated with quinolone resistance in *H. pylori* [49,51]. Notably, *H. pylori* lacks genes encoding topoisomerase IV (encoded by *parC* and *parE* genes), which is targeted by quinolones in other bacteria [52]. Moreover, active efflux pumps seem not to be involved in mediating fluoroquinolone resistance [53].

In our study, *H. pylori* strains with a rifampicin susceptible phenotype (i.e., MICs ≤ 4 mg/L) showed no mutation in the RRDR of *rpoB*. Amino acid substitutions, L525P and H540N, were associated with resistance and MICs of >32 mg/L. Noteworthy, application of the current EUCAST clinical breakpoint for rifampicin of 1 mg/L would split the wild-type population (Figure 1). This in turn introduces serious interpretation problems, since some *H. pylori* strains that do not carry point mutations in the RRDR of *rpoB* would be classified as rifampicin resistant, representing a major error. Given the rifampicin MIC distribution of *H. pylori* isolates analysed in this study, we recommend using the clinical breakpoint of 4 mg/L proposed by Hays et al. [22] and the CASFM/EUCAST guidelines [29].

Findings gained in this study can be useful for future research that may aim at performing culture-independent WGS directly from clinical specimens for the prediction of phenotypic drug susceptibility in *H. pylori*. Some studies have already successfully applied WGS directly on gastric biopsies for the detection of *H. pylori* [54,55]. Moreover, in-house developed and commercial protocols (e.g., [56,57]) are becoming available for the depletion of human DNA or the enrichment of bacterial DNA prior to performing WGS, thereby increasing the efficiency and cost-effectiveness of NGS due to less human DNA background in samples.

Our study has several limitations: It was designed as a single centre laboratory-based study using clinical *H. pylori* isolates. For some antibiotics, there were only a few (i.e., rifampicin) or no resistant *H. pylori* isolates (i.e., tetracycline) available. Thus, the analytical sensitivity of WGS in determining phenotypic rifampicin resistance based on mutations in the RRDR of *rpoB* might have been overestimated as rifampicin resistance has also been reported in *H. pylori* isolates lacking these mutations [8]. For tetracycline, calculation of the analytical sensitivity of WGS was not possible due to a lack of resistant *H. pylori* isolates. Moreover, genotype-based prediction of tetracycline resistance in *H. pylori* is additionally hampered since isolates without mutations at nucleotide positions, 926 to 928, in the 16S rRNA gene display a resistant phenotype [58]. Therefore, tetracycline resistance seems to be multifactorial, involving alterations in ribosomal binding, enzymatic degradation of antibiotics, a reduction of membrane permeability, and an active efflux [59,60,61].

In this study, we have not examined the possible role of active efflux mechanisms. Their involvement in intrinsic antibiotic resistance in *H. pylori* is still a matter of debate and would require transcriptional analyses of the genes encoding drug efflux systems (e.g., by using a meta-transcriptomics approach), which was beyond the scope of this study.

## 5. Conclusions

It is evident that increasing antimicrobial resistance represents a significant challenge in the successful management of *H. pylori* infections. This highlights the importance of rapid determination of drug susceptibility in tailoring treatments to increase *H. pylori* eradication success. In addition to culture based phenotypic DST, high-throughput sequencing is rapidly changing the landscape in medical microbiology. In this study, we could demonstrate that genetic determinants identified by WGS in the *H. pylori* genome are significantly correlated to phenotypic drug resistance. This allows the prediction of phenotypic clarithromycin, rifamycin, and levofloxacin resistance based on genotypic information in the 23S rRNA, *gyrA*, and *rpoB* genes with high specificity and good sensitivity in a clinically relevant timeframe.

## Figures and Tables

**Table 1:** Point mutations identified by WGS in the 23S rRNA gene at nucleotide positions, A2146 and A2147, in 96 clarithromycin resistant *H. pylori* isolates and their corresponding MICs.

Number of Clinical *H. pylori* Strains	MIC of Clarithromycin (mg/L)	23S rRNA Mutation ^a^
1	>256	A2146C
28	8 to 256	A2146G
67	1.5 to 256	A2147G

^a^ Nucleotide exchange relative to *H. pylori* reference strain 26695 sequence. WGS: whole genome sequencing MICs: minimum inhibitory concentrations.

**Table 2:** Agreement between phenotypic DST results and identified point mutations in the 23S rRNA gene at nucleotide positions, A2146 and A2147, conferring resistance to macrolides (*N* = 140).

		Phenotypic DST	
		Susceptible	Resistant
WGS	Susceptible ^a^	43	1
	Resistant ^b^	0	96

^a^ no mutation present at nucleotide positions, A2146 and A2147, in the 23S rRNA gene. ^b^ A mutation present at nucleotide positions, A2146 and A2147, in the 23S rRNA gene. DST: drug susceptibility testing.

**Table 3:** Amino acid exchanges identified by WGS in the QRDR of the *gyrA* gene at codon 87 and/or 91 in 46 levofloxacin resistant *H. pylori* isolates and their corresponding MICs.

Number of Clinical *H. pylori* Strains	MIC of Levofloxacin (mg/L)	*gyrA* aa Exchange ^a^
1	>32	N87I
22	>32	N87K
2	4 to 32	N87Y
8	16 to 32	D91G
5	4 to 32	D91N
6	4 to 32	D91Y
2	>32	N87K D91N

^a^ Amino acid exchange relative to *H. pylori* reference strain 26,695 sequence. QRDR: Quinolone resistance determinig region.

**Table 4:** Agreement between phenotypic DST results and identified aa exchanges in the *gyrA* gene at codon 87 and/or 91 conferring resistance to fluoroquinolones (*N* = 138).

		Phenotypic DST	
		Susceptible	Resistant
WGS	Susceptible ^a^	91	1
	Resistant ^b^	0	46

^a^ no aa exchange present in the QRDR of the *gyrA* gene at codon 87 and/or 91. ^b^ An aa exchange present in the QRDR of the *gyrA* gene at codon 87 and/or 91.

**Table 5:** Amino acid exchanges identified by WGS in the RRDR of the *rpoB* gene conferring resistance to rifamycins and their corresponding MICs.

Number of Clinical *H. pylori* Strains	MIC of Levofloxacin (mg/L)	*rpoB* aa Exchange ^a^
2	>32	H540N
2	>32	L525P

^a^ Amino acid exchange relative to *H. pylori* reference strain 26,695 sequence.

**Table 6:** Agreement between phenotypic DST results and identified aa exchanges in the RRDR of the *rpoB* gene conferring resistance to rifamycins (*N* = 140).

		Phenotypic DST	
		Susceptible	Resistant
WGS	Susceptible ^a^	136	0
	Resistant ^b^	0	4

^a^ No aa exchange present in the RRDR of the *rpoB* gene. ^b^ An aa exchange present in the RRDR of the *rpoB* gene.

**Table 7:** Point mutations identified by WGS in the primary binding site of tetracycline in the 16S rRNA gene at nucleotide positions, 926 to 928, and their corresponding MICs.

Number of Clinical *H. pylori* Strains	MIC of Levofloxacin (mg/L)	16S rRNA Mutation ^a^
2	0.25 to 0.5	A926T
1	0.032	A926G
1	0.125	A928C

^a^ Nucleotide exchange relative to *H. pylori* reference strain 26,695 sequence.

## Supplementary Materials

The following are available online at http://www.mdpi.com/2077-0383/8/1/53/s1. Table S1. *H. pylori* isolates showing discrepant phenotypic DST and WGS results were sent to the *H. pylori* reference centre in Bordeaux, France, for culture based phenotypic DST. Table S2. Comparison of frameshift mutations and SNPs detected in the frxA gene of metronidazole susceptible and resistant *H. pylori* isolates. Table S3. Comparison of frameshift mutations and truncations detected in the *rdxA* gene of metronidazole susceptible and resistant *H. pylori* isolates. Table S4. Comparison of SNPs detected in the *rdxA* gene of metronidazole susceptible and resistant *H. pylori* isolates. Table S5. List of all frameshift mutations and SNPs detected in the *frxA* gene of metronidazole susceptible and resistant *H. pylori* isolates. Table S6. List of all frameshift mutations and SNPs detected in the *rdxA* gene of metronidazole susceptible and resistant *H. pylori* isolates. Table S7. List of all SNPs detected in the *gyrA* and *gyrB* genes of the discrepant *H. pylori* isolate showing a levofloxacin resistant phenotype. Figure S1. Association between phenotypic drug resistance and mutations in the 23S rRNA gene at nucleotide positions A2146 and A2147 (panel A) and mutations in the *gyrA* QRDR (panel B). One discrepant *H. pylori* isolate showing phenotypic levofloxacin resistance without mutations in the *gyrA* QRDR is displayed in red. Availability of Data and Material: Whole genome sequences of the *H. pylori* strains analyzed in this study are available on NCBI under accession numbers.
