# Supplementary material for: Genetic Determinants and Prediction of Antibiotic Resistance Phenotypes in Helicobacter pylori
Source: J Clin Med. 2019 Jan 7;8(1):53. doi: 10.3390/jcm8010053 (PMC6351930; doi:10.3390/jcm8010053)
Supplement: Supplementary file 1 [file jcm-08-00053-s001.docx]

**Supplementary Information**

Article

Genetic Determinants and Prediction of Antibiotic Resistance Phenotypes in *Helicobacter pylori*

Francis N. Lauener ^1,†^, Frank Imkamp ^1,†^, Philippe Lehours ^2,3^, Alice Buissonnière ^2,3^,
Lucie Benejat ^2,3^, Reinhard Zbinden ^1^, Peter M. Keller ^1,4^ and Karoline Wagner ^1,^*^,‡^

^1^ Institute of Medical Microbiology, University of Zurich, 8006 Zurich, Switzerland; francisnicolai.lauener@uzh.ch (F.N.L.); imkamp@imm.uzh.ch (K.I.); rzbinden@imm.uzh.ch (R.Z.); pkeller@imm.uzh.ch (P.M.K.)

^2^ INSERM UMR1053, Bordeaux Research in Translational Oncology, BaRITOn, Université de Bordeaux, 33076 Bordeaux, France; philippe.lehours@u-bordeaux.fr (P.L.); alice.buissonniere@chu-bordeaux.fr (A.B.); lucie.bruhl@chu-bordeaux.fr (L.B.)

^3^ French National Reference Centre for *Campylobacter* and *Helicobacter*, Bordeaux Hospital, 33076 Bordeaux, France

^4^ Institute for Infectious Diseases, University of Bern, 3001 Bern, Switzerland

***** Correspondence: karoline.wagner@usb.ch; Tel.: +41612073262

† These authors contributed equally to the manuscript

‡ Current affiliation: Division of Infection Diagnostics, Department of Biomedicine, University of Basel, Petersplatz 10, 4051 Basel, Switzerland

Received: 5 December 2018; Accepted: 31 December 2018; Published: date

**Supplementary Figures**


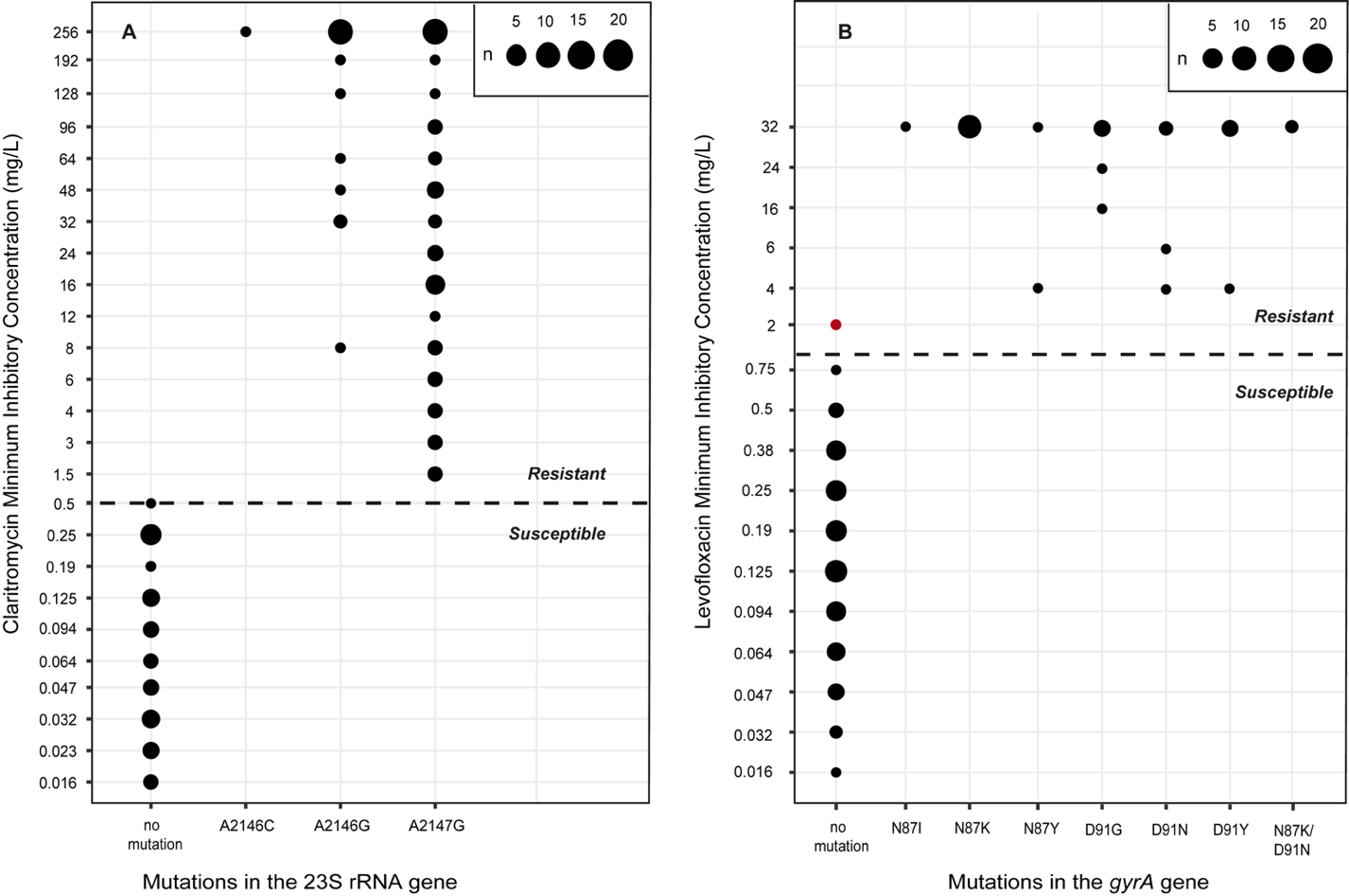


Figure S1. Association between phenotypic drug resistance and mutations in the 23S rRNA gene at nucleotide positions A2146 and A2147 (panel A) and mutations in the *gyrA* QRDR (panel B). One discrepant *H. pylori* isolate showing phenotypic levofloxacin resistance without mutations in the *gyrA* QRDR is displayed in red.

**Supplementary Tables**

Table S1. *H. pylori* isolates showing discrepant phenotypic DST and WGS results were sent to the *H. pylori* reference centre in Bordeaux, France, for culture based phenotypic DST.

|  | **MIC (mg/L)** | |  | **WGS** | |
| --- | --- | --- | --- | --- | --- |
| **Drug** | **IMM** | **Bordeaux** |  | **Gene** | **Mutation** |
| Clarithromycin | 0.5 | 0.5 and >256 |  | 23S rRNA | A2146G |
|  | 0.5 | 0.19 and 6 |  | 23S rRNA | A2147G |
|  | 1.5 | 0.5 |  | 23S rRNA | T2182 |
| Levofloxacin | 2.0 | 1.5 |  | *gyrA* | - |
|  | 2.0 | nd |  | *gyrA* | - |
|  | 4.0 | nd |  | *gyrA* | - |
| nd these two *H. pylori* strains could not be regrown and were subsequently not sent  for discrepancy analysis to the *H. pylori* reference centre in Bordeaux | | | | | |

Table S2. Comparison of frameshift mutations and SNPs detected in the *frxA* gene of metronidazole susceptible and resistant *H. pylori* isolates.

| **Gene** | **Codon^1^** | **Number of susceptible**  ***H. pylori* isolates** |  | **Number of resistant *H. pylori* isolates** | **Reference** |
| --- | --- | --- | --- | --- | --- |
| *frxA* | 105 (frameshift) | - |  | 1 | Frameshifts in *frxA* suggested to confer  metronidazole resistance [4] |
|  | 149 (frameshift) | - |  | 1 |  |
|  | 192 (frameshift) | - |  | 1 |  |
|  | 18 (frameshift) | 14 |  | 46 | These frameshifts have not been previously reported in the literature |
|  | A16T | 3 |  | 5 |  |
|  | A32V | 2 |  | 3 |  |
|  | A85V | - |  | 3 |  |
|  | I44F | - |  | 3 |  |
|  | I117M | 1 |  | 3 |  |
|  | M126F | 1 |  | 2 |  |
|  | S43A | - |  | 3 |  |
|  | V7I | 2 |  | 3 |  |
|  | Y62D | 4 |  | 5 |  |
| ^1^ Just amino acid exchanges and frameshift mutations that occurred more frequently in metronidazole resistant  than in susceptible *H. pylori* isolates are displayed. | | | | | |

Table S3. Comparison of frameshift mutations and truncations detected in the *rdxA* gene of metronidazole susceptible and resistant *H. pylori* isolates.

| **Gene** | **Codon^1^** | **Number of susceptible *H. pylori* isolates** | **Number of resistant *H. pylori* isolates** | **Reference** |
| --- | --- | --- | --- | --- |
| *rdxA* | 18  (frameshift) | - | 2 | Frameshifts in *rdxA*  suggested to confer  metronidazole resistance  [4] |
|  | 38  (frameshift) | - | 1 |  |
|  | 112 (frameshift) | - | 1 |  |
|  | 190 (frameshift) | - | 1 |  |
|  | 50  (truncation) | - | 2 | truncation leads to  metronidazole resistance [5] |
|  | 31  (truncation) | - | 1 | Truncations in *rdxA*  suggested to lead to  metronidazole resistance [5] |
|  | 119 (truncation) | - | 1 |  |
|  | 146 (truncation) | - | 1 |  |
|  | 96  (frameshift) | 3 | 4 | These frameshifts have not been previously reported in the literature |
|  | 62  (frameshift) | 1 | 3 |  |
|  | 162 (frameshift) | 1 | 1 |  |
| ^1^ Just frameshift mutations that occurred more frequently in metronidazole resistant than in  susceptible *H. pylori* isolates are displayed. | | | | |

Table S4. Comparison of SNPs detected in the *rdxA* gene of metronidazole susceptible and resistant *H. pylori* isolates.

| **Gene** | **Codon^1^** |  | **Number of susceptible *H. pylori* isolates** |  | **Number of resistant *H. pylori* isolates** | **Reference** |
| --- | --- | --- | --- | --- | --- | --- |
| *rdxA* | R16C |  | - |  | 10 | R16C occurs only in metronidazole resistant *H. pylori* strains [6-8] |
|  | K64N |  | - |  | 6 | K64N occurs solely in metronidazole resistant *H. pylori* strains [9] |
|  | P106S |  | - |  | 6 | P106S occurs solely in metronidazole resistant *H. pylori* strains [9] |
|  | A67V |  | - |  | 5 | A67V occurs solely in metronidazole resistant *H. pylori* strains [10] |
|  | A68E |  | - |  | 3 | A68T occurs solely in metronidazole resistant *H. pylori* strains [9] |
|  | D59N |  | 55 |  | 81 | These mutations have been previously reported in metronidazole susceptible and resistant *H. pylori* strains [9] |
|  | R90K |  | 19 |  | 37 |  |
|  | R131K |  | 28 |  | 33 |  |
|  | T31E |  | 19 |  | 30 |  |
|  | A118T |  | 16 |  | 22 |  |
|  | H97Y |  | 8 |  | 14 |  |
|  | G98S |  | 11 |  | 10 |  |
|  | A68V |  | 5 |  | 8 |  |
|  | L62V |  | 4 |  | 7 |  |
|  | M56I |  | 4 |  | 7 |  |
|  | R16H |  | 2 |  | 7 |  |
|  | V204I |  | 3 |  | 4 |  |
|  | M56V |  | - |  | 2 |  |
|  | V111A |  | 2 |  | 5 | These SNPs have not been previously reported in the literature |
|  | A183V |  | 5 |  | 9 |  |
|  | M21T |  | - |  | 3 |  |
|  | P44L |  | - |  | 3 |  |
|  | Q197K |  | 2 |  | 3 |  |
|  | S30R |  | 2 |  | 3 |  |
|  | S45R |  | - |  | 3 |  |
|  | A206T |  | - |  | 2 |  |
|  | H25R |  | - |  | 2 |  |
|  | L185Y |  | - |  | 2 |  |
|  | L188V |  | - |  | 2 |  |
| ^1^ Just amino acid exchanges and frameshift mutations that occurred more frequently in metronidazole resistant  than in susceptible *H. pylori* isolates are displayed. | | | | | | |

Table S5. List of all frameshift mutations and SNPs detected in the *frxA* gene of metronidazole susceptible and resistant *H. pylori* isolates.

| **Gene** | **Codon** |  | **MIC**  **≤8 mg/L^1^** | **MIC**  **>8 mg/L^2^** | |
| --- | --- | --- | --- | --- | --- |
| *frxA* | 18 | Frameshift | 0.016 | 256 | |
|  | 105 | Frameshift | - | 256 | |
|  | 149 | Frameshift | - | 256 | |
|  | 192 | Frameshift | - | 24 | |
|  | A153V | Amino acid exchange | - | 256 | |
|  | A15V | Amino acid exchange | - | 256 | |
|  | A16T | Amino acid exchange | 0.016 | 256 | |
|  | A32V | Amino acid exchange | - | 256 | |
|  | A40S | Amino acid exchange | 0.38 | 256 | |
|  | A67V | Amino acid exchange | 1.5 | - | |
|  | A70V | Amino acid exchange | 0.5 | - | |
|  | A85V | Amino acid exchange | 16 | 128 | |
|  | C193S | Amino acid exchange | 0.064 | 256 | |
|  | C19T | Amino acid exchange | - | 256 | |
|  | D109G | Amino acid exchange | - | 24 | |
|  | D109N | Amino acid exchange | - | 32 | |
|  | D94N | Amino acid exchange | 0.38 | - | |
|  | E164K | Amino acid exchange | - | 256 | |
|  | E169K | Amino acid exchange | - | 256 | |
|  | E176K | Amino acid exchange | 0.064 | 256 | |
|  | E177K | Amino acid exchange | 0.5 | - | |
|  | E75D | Amino acid exchange | 0.19 | 256 | |
|  | F72S | Amino acid exchange | 0.064 | 256 | |
|  | G73S | Amino acid exchange | 0.064 | 256 | |
|  | G76A | Amino acid exchange | 0.094 | 256 | |
|  | I117M | Amino acid exchange | 0.75 | 256 | |
|  | I118F | Amino acid exchange | 0.38 | - | |
|  | I144V | Amino acid exchange | 1 | - | |
|  | I44F | Amino acid exchange | 24 | 256 | |
|  | K208N | Amino acid exchange | 0.75 | 24 | |
|  | M155I | Amino acid exchange | 0.064 | - | |
|  | N111D | Amino acid exchange | 0.064 | 256 | |
|  | N111H | Amino acid exchange | - | 16 | |
|  | N124S | Amino acid exchange | 0.064 | 256 | |
|  | N182D | Amino acid exchange | 0.75 | - | |
|  | Q27E | Amino acid exchange | 32 | 256 | |
|  | R38K | Amino acid exchange | 0.25 | 0.38 | |
|  | R86Q | Amino acid exchange | 0.25 | - | |
|  | S43A | Amino acid exchange | 24 | 256 | |
|  | S93N | Amino acid exchange | 0.75 | - | |
|  | T110A | Amino acid exchange | 0.064 | - | |
|  | V34I | Amino acid exchange | 0.38 | 256 | |
| **Gene** | **Codon** |  | **MIC**  **≤8 mg/L^1^** | **MIC**  **>8 mg/L^2^** | |
| *frxA* | V6I | Amino acid exchange | 1 | - | |
|  | V7I | Amino acid exchange | 0.19 | 256 | |
|  | V7L | Amino acid exchange | 0.064 | - | |
|  | V96I | Amino acid exchange | 0.19 | - | |
| ^1^ If the same amino acid exchange was identified in multiple *H. pylori* strains with a  metronidazole susceptible phenotype, the lowest MIC is displayed  ^2^ If the same amino acid exchange was identified in multiple *H. pylori* strains with a  metronidazole resistant phenotype, the highest MIC is displayed | | | | |  |
|  |  |  |  |  | |

Table S6. List of all frameshift mutations and SNPs detected in the *rdxA* gene of metronidazole susceptible and resistant *H. pylori* isolates.

| **Gene** | **Codon** |  | **MIC**  **≤8 mg/L^1^** | **MIC**  **>8 mg/L^2^** | |
| --- | --- | --- | --- | --- | --- |
| *rdxA* | 18 | Frameshift | - | 256 | |
|  | 38 | Frameshift | - | 256 | |
|  | 43 | Frameshift | - | 256 | |
|  | 61 | Frameshift | - | 256 | |
|  | 62 | Frameshift | - | 256 | |
|  | 75 | Frameshift | - | 256 | |
|  | 96 | Frameshift | 0.094 | 256 | |
|  | 97 | Frameshift | 0.19 | 256 | |
|  | 112 | Frameshift | - | 256 | |
|  | 138 | Frameshift | 0.016 | 256 | |
|  | 162 | Frameshift | 0.5 | 256 | |
|  | 190 | Frameshift |  | 256 | |
|  | 50 | Truncation | 0.094 | 256 | |
|  | 119 | Truncation | - | 256 | |
|  | 146 | Truncation | - | 256 | |
|  | A118S | Amino acid exchange | 0.016 | 256 | |
|  | A118T | Amino acid exchange | 0.016 | 256 | |
|  | A183V | Amino acid exchange | 0.016 | 256 | |
|  | A193T | Amino acid exchange | - | 256 | |
|  | A195V | Amino acid exchange | - | 256 | |
|  | A206T | Amino acid exchange | - | 256 | |
|  | A299G | Amino acid exchange | - | 256 | |
|  | A37V | Amino acid exchange | - | 256 | |
|  | A465G | Amino acid exchange | 0.5 | - | |
|  | A633G | Amino acid exchange | - | 256 | |
|  | A67V | Amino acid exchange | - | 256 | |
|  | A68E | Amino acid exchange | - | 256 | |
|  | A68T | Amino acid exchange | 0.064 | 256 | |
|  | A68V | Amino acid exchange | 0.125 | 256 | |
|  | A80T | Amino acid exchange | 0.38 | 256 | |
|  | A82S | Amino acid exchange | 0.19 | - | |
|  | A91G | Amino acid exchange | - | 256 | |
|  | C131T | Amino acid exchange | - | 256 | |
|  | C140F | Amino acid exchange | - | 256 | |
|  | C140Y | Amino acid exchange | 3 | 256 | |
|  | C148T | Amino acid exchange | - | 256 | |
|  | C19Y | Amino acid exchange | - | 256 | |
|  | C378T | Amino acid exchange | 0.5 | - | |
|  | C381T | Amino acid exchange | 0.5 | - | |
|  | C46T | Amino acid exchange | - | 256 | |
|  | C92A | Amino acid exchange | - | 256 | |
|  | D205A | Amino acid exchange | - | 256 | |
| **Gene** | **Codon** |  | **MIC**  **≤8 mg/L^1^** | **MIC**  **>8 mg/L^2^** | |
| *rdxA* | D59N | Amino acid exchange | 0.016 | 256 | |
|  | D5N | Amino acid exchange | 0.064 | - | |
|  | D61N | Amino acid exchange | - | 256 | |
|  | E175Q | Amino acid exchange | 0.25 | 256 | |
|  | E194G | Amino acid exchange | - | 256 | |
|  | E27T | Amino acid exchange | 0.25 | - | |
|  | E34G | Amino acid exchange | 0.75 | - | |
|  | E74G | Amino acid exchange | - | 256 | |
|  | F3I | Amino acid exchange | - | 256 | |
|  | G122S | Amino acid exchange | 0.75 | 256 | |
|  | G123A | Amino acid exchange | 3 | - | |
|  | G163V | Amino acid exchange | - | 256 | |
|  | G170S | Amino acid exchange | 0.016 | 256 | |
|  | G189C | Amino acid exchange | - | 256 | |
|  | G225A | Amino acid exchange | - | 256 | |
|  | G392A | Amino acid exchange | - | 256 | |
|  | G434A | Amino acid exchange | - | 256 | |
|  | G476A | Amino acid exchange | - | 256 | |
|  | G47A | Amino acid exchange | 0.19 | 256 | |
|  | G504A | Amino acid exchange | 0.5 | - | |
|  | G523C | Amino acid exchange | 0.5 | - | |
|  | G618A | Amino acid exchange | - | 256 | |
|  | G98S | Amino acid exchange | 0.064 | 256 | |
|  | H25R | Amino acid exchange | - | 256 | |
|  | H53R | Amino acid exchange | 0.064 | 256 | |
|  | H53Y | Amino acid exchange | 0.25 | - | |
|  | H69R | Amino acid exchange | - | 256 | |
|  | H69Y | Amino acid exchange | 0.125 | - | |
|  | H97Y | Amino acid exchange | 0.016 | 256 | |
|  | K110R | Amino acid exchange | 0.016 | - | |
|  | K179R | Amino acid exchange | 0.125 | - | |
|  | K60R | Amino acid exchange | - | 24 | |
|  | K64N | Amino acid exchange | 3 | 256 | |
|  | K64R | Amino acid exchange | 1 | - | |
|  | L121F | Amino acid exchange | - | 256 | |
|  | L167S | Amino acid exchange | - | 82 | |
|  | L185Y | Amino acid exchange | - | 256 | |
|  | L188V | Amino acid exchange | - | 256 | |
|  | L62V | Amino acid exchange | 0.064 | 256 | |
|  | M56I | Amino acid exchange | 0.094 | 256 | |
|  | M56V | Amino acid exchange | - | 256 | |
|  | M84I | Amino acid exchange | 0.75 | - | |
|  | P106L | Amino acid exchange | - | 256 | |
|  | P106S | Amino acid exchange | 0.5 | 256 | |
|  | P115L | Amino acid exchange | - | 256 | |
|  | P166L | Amino acid exchange | - | 256 | |
| **Gene** | **Codon** |  | **MIC**  **≤8 mg/L^1^** | **MIC**  **>8 mg/L^2^** | |
| *rdxA* | P44L | Amino acid exchange | - | 256 | |
|  | P44S | Amino acid exchange | 0.96 | - | |
|  | P91S | Amino acid exchange | 0.25 | - | |
|  | Q139K | Amino acid exchange | - | 256 | |
|  | Q139R | Amino acid exchange | - | 256 | |
|  | Q197K | Amino acid exchange | 0.25 | 256 | |
|  | Q6H | Amino acid exchange | 0.125 | 256 | |
|  | R131K | Amino acid exchange | 0.016 | 256 | |
|  | R16C | Amino acid exchange | - | 256 | |
|  | R176H | Amino acid exchange | - | 256 | |
|  | R41K | Amino acid exchange | - | 256 | |
|  | R90K | Amino acid exchange | 0.016 | 256 | |
|  | S108A | Amino acid exchange | 0.25 | 128 | |
|  | S30R | Amino acid exchange | 0.016 | 256 | |
|  | S45R | Amino acid exchange | - | 256 | |
|  | S81L | Amino acid exchange | - | 256 | |
|  | S88P | Amino acid exchange | 0.064 | 256 | |
|  | T31A | Amino acid exchange | 1 | - | |
|  | T31E | Amino acid exchange | 0.016 | 256 | |
|  | T58A | Amino acid exchange | 0.5 | - | |
|  | V111A | Amino acid exchange | 0.5 | 256 | |
|  | V111I | Amino acid exchange | - | 256 | |
|  | V172I | Amino acid exchange | 0.064 | 256 | |
|  | V204I | Amino acid exchange | 0.25 | 256 | |
| ^1^ If the same amino acid exchange was identified in multiple *H. pylori* strains with a  metronidazole susceptible phenotype, the lowest MIC is displayed  ^2^ If the same amino acid exchange was identified in multiple *H. pylori* strains with a  Metronidazole resistant phenotype, the highest MIC is displayed | | | | |  |

Table S7. List of all SNPs detected in the *gyrA* and *gyrB* genes of the discrepant *H. pylori* isolate showing a levofloxacin resistant phenotype.

| **Discrepant *H. pylori* isolate** | **Levofloxacin MIC (mg/L)** | |  | **WGS** | |
| --- | --- | --- | --- | --- | --- |
|  | **IMM Zurich** | **Bordeaux** |  | **Amino acid exchanges**  **in *gyrA*** | **Amino acid exchanges**  **in *gyrB*** |
| *H. pylori* isolate 1 | 2.0 | 1.5 |  | M191I | T149I |
|  |  |  |  | V199A | I238V |
|  |  |  |  | G208E | A283T |
|  |  |  |  | I234V | G298D |
|  |  |  |  | H354R | R349H |
|  |  |  |  | R397Q | R484K |
|  |  |  |  | N495S | S546G |
|  |  |  |  | I498T | R579H |
|  |  |  |  | Y513H | L601F |
|  |  |  |  | A524V | T644A |
|  |  |  |  | D610N | N669H |
|  |  |  |  | E632G | H698Y |
|  |  |  |  | S633G |  |
|  |  |  |  | S652G |  |
|  |  |  |  | E679D |  |
|  |  |  |  | K694R |  |
|  |  |  |  | G733E |  |
|  |  |  |  | M803V |  |
|  |  |  |  | A272G |  |
|  |  |  |  | G849A |  |

**Availability of Data and Material:** Whole genome sequences of the *H. pylori* strains analysed in this study are available on NCBI under accession numbers:

RJDZ00000000, RJEA00000000, RJEB00000000, RJEC00000000, RJED00000000, RJEE00000000, RJEF00000000, RJEG00000000, RJEH00000000, RJEI00000000, RJEJ00000000, JEL00000000, RJEM00000000, RJEN00000000, RJEO00000000,

RJEP00000000, RJEQ00000000, RJER00000000, RJES00000000, RJET00000000, RJEU00000000, RJEV00000000, RJEW00000000, RJEX00000000, RJEY00000000,

RJEZ00000000, RJFA00000000, RJFB00000000, RJFC00000000, RJFD00000000,

RJFE00000000, RJFF00000000, RJFG00000000, RJFH00000000, RJFI00000000,

RJFJ00000000, RJFK00000000, RJFL00000000, RJFM00000000, RJFN00000000,

RJFO00000000, RJFP00000000, RJFQ00000000, RJFR00000000, RJFS00000000,

RJFT00000000, RJFU00000000, RJFV00000000, RJFW00000000, RJFX00000000,

RJFY00000000, RJFZ00000000, RJGA00000000, RJGB00000000, RJGC00000000,

RJGD00000000, RJGE00000000, RJGF00000000, RJGG00000000, RJGH00000000,

RJGI00000000, RJGJ00000000, RJGK00000000, RJGL00000000, RJGM00000000,

RJGN00000000, RJGO00000000, RJGP00000000, RJGQ00000000, RJGR00000000,

RJGS00000000, RJGT00000000, RJGU00000000, RJGV00000000, RJGW00000000,

RJGX00000000, RJGY00000000, RJGZ00000000, RJHA00000000, RJHB00000000,

RJHC00000000, RJHD00000000, RJHE00000000, RJHF00000000, RJHG00000000,

RJHH00000000RJHI00000000, RJHJ00000000, RJHK00000000, RJHL00000000,

RJHM00000000, RJHN00000000, RJHO00000000, RJHP00000000, RJHQ00000000,

RJHR00000000, RJHS00000000, RJHT00000000, RJHU00000000, RJHV00000000,

RJHW00000000, RJHX00000000, RJHY00000000, RJHZ00000000, RJIA00000000,

RJIB00000000, RJIC00000000, RJID00000000, RJIE00000000, RJIF00000000,

RJIG00000000, RJIH00000000, RJII00000000, RJIJ00000000, RJIK00000000,

RJIL00000000, RJIM00000000, RJIN00000000, RJIO00000000, RJIP00000000,

RJIQ00000000, RJIR00000000, RJIS00000000, RJIT00000000, RJIU00000000,

RJIV00000000, RJIW00000000, RJIX00000000, RJIY00000000, RJIZ00000000.

**References**

1. EUCAST. Breakpoint tables for interpretation of MICs and zone diameters. Version 8.0. http://www.eucast.org. 2018.
2. CASFM/EUCAST. http://www.sfm-microbiologie.org/UserFiles/ files/casfm/CASFMV1_0_MARS_2018.pdf. 2018.

3. Hays C, Burucoa C, Lehours P, Tran CT, Leleu A, Raymond J. Molecular characterization of *Helicobacter pylori* resistance to rifamycins. Helicobacter. 2018, 23:e12451.

4. Masaoka T, Suzuki H, Kurabayashi K, Nomoto Y, Nishizawa T, Mori M, et al., Could frameshift mutations in the *frxA* and *rdxA* genes of *Helicobacter pylori* be a marker for metronidazole resistance? Aliment Pharmacol Ther Symposium Series. 2006: Wiley Online Library; 2006: 81-7.

5. Lee SM, Kim N, Kwon YH, Nam RH, Kim JM, Park JY, et al., *rdxa, frxa*, and efflux pump in metronidazole‐resistant *Helicobacter pylori*: Their relation to clinical outcomes. J Gastroenterol Hepatol. 2018, 33:681-8.

6. Marais A, Bilardi C, Cantet F, Mendz GL, Mégraud F. Characterization of the genes *rdxA* and *frxA* involved in metronidazole resistance in *Helicobacter pylori*. Res Microbiol. 2003, 154:137-44.

7. Jeong JY, Mukhopadhyay AK, Dailidiene D, Wang Y, Velapatiño B, Gilman RH, et al., Sequential inactivation of *rdxA* (HP0954) and *frxA* (HP0642) nitroreductase genes causes moderate and high-level metronidazole resistance in *Helicobacter pylori*. J Bacteriol. 2000, 182:5082-90.

8. Binh TT, Suzuki R, Trang TTH, Kwon DH, Yamaoka Y. Search for novel candidate mutations for metronidazole resistance in *Helicobacter pylori* using next-generation sequencing. Antimicrob Agents Chemother. 2015, 59:2343-8.

9. Goodwin A, Kersulyte D, Sisson G, Veldhuyzen van Zanten SJ, Berg DE,et al., Metronidazole resistance in *Helicobacter pylori* is due to null mutations in a gene (*rdxA*) that encodes an oxygen‐insensitive NADPH nitroreductase. Mol Microbiol. 1998, 28:383-93.

10. Tankovic J, Lamarque D, Delchier JC, Soussy CJ, Labigne A, Jenks PJ. Frequent association between alteration of the *rdxA* gene and metronidazole resistance in french and north African isolates of *Helicobacter pylori*. Antimicrob Agents Chemother. 2000, 44:608-13.
